# Development of caecaloids to study host–pathogen interactions: new insights into immunoregulatory functions of *Trichuris muris* extracellular vesicles in the caecum

**DOI:** 10.1016/j.ijpara.2020.06.001

**Published:** 2020-08

**Authors:** María A. Duque-Correa, Fernanda Schreiber, Faye H. Rodgers, David Goulding, Sally Forrest, Ruby White, Amy Buck, Richard K. Grencis, Matthew Berriman

**Affiliations:** a Wellcome Sanger Institute, Wellcome Genome Campus, Hinxton CB10 1SA, UK; b Institute of Immunology & Infection Research, School of Biological Sciences, University of Edinburgh, Edinburgh EH9 3FL, UK; c Lydia Becker Institute of Immunology and Inflammation, Wellcome Trust Centre for Cell Matrix Research and Faculty of Biology, Medicine and Health, University of Manchester, Manchester M13 9PT, UK

**Keywords:** Organoids, Caecum, Caecaloids, Intestinal epithelial cells, *Trichuris muris*, Extracellular vesicles, Immunoregulation

## Abstract

The caecum, an intestinal appendage in the junction of the small and large intestines, displays a unique epithelium that serves as an exclusive niche for a range of pathogens including whipworms (*Trichuris* spp.). While protocols to grow organoids from small intestine (enteroids) and colon (colonoids) exist, the conditions to culture organoids from the caecum have yet to be described. Here, we report methods to grow, differentiate and characterise mouse adult stem cell-derived caecal organoids, termed caecaloids. We compare the cellular composition of caecaloids with that of enteroids, identifying differences in intestinal epithelial cell populations that mimic those found in the caecum and small intestine. The remarkable similarity in the intestinal epithelial cell composition and spatial conformation of caecaloids and their tissue of origin enables their use as an in vitro model to study host interactions with important caecal pathogens. Thus, exploiting this system, we investigated the responses of caecal intestinal epithelial cells to extracellular vesicles secreted/excreted by the intracellular helminth *Trichuris muris.* Our findings reveal novel immunoregulatory effects of whipworm extracellular vesicles on the caecal epithelium, including the downregulation of responses to nucleic acid recognition and type-I interferon signalling.

## Highlights

• Development of new methods to generate, culture and characterise mouse caecaloids is described.
• Caecaloids recapitulate the caecal epithelium composition and spatial organisation.
• Caecaloids can be used to study host–caecal pathogen interactions in vitro.
• *Trichuris muris* EVs exert novel immunoregulatory effects on intestinal epithelial cells.

## Graphical abstract

**Figure:**
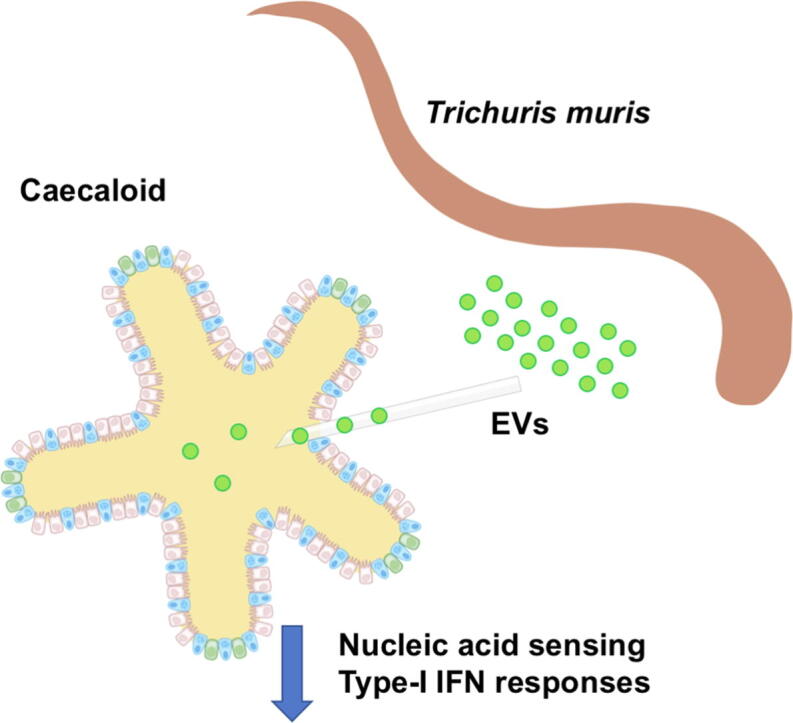

## 1 Introduction

The intestine is a continuous tube that stretches from the pylorus to the anus, lined internally by a monolayer of columnar epithelium (Mowat and Agace, 2014). Although continuous, the intestine is composed of defined segments with distinct macro- and microscopic appearances, and specialised functions (Mowat and Agace, 2014, Nguyen et al., 2015). These segments are the duodenum, jejunum and ileum of the small intestine, and caecum, proximal, transverse and distal colon, rectum and anus of the large intestine (Mowat and Agace, 2014, Nguyen et al., 2015).

The caecum is an intestinal appendage at the junction of the small intestine and the large intestine (Burns et al., 2004). This blind-ended sac harbours commensal bacteria that in humans can replenish gut microbiota after disturbances and in the mouse are involved in the fermentative digestion of plant polysaccharides that cannot be digested by enzymes of the small intestine (Burns et al., 2004, Backhed et al., 2005, Eckburg et al., 2005, Al Alam et al., 2012, Mowat and Agace, 2014, Nguyen et al., 2015). Microscopically, the caecum is different from the small intestine because it lacks villi and is more similar to the colon since its mucosa consists of crypts of Lieberkühn with only short regions of flat surface epithelium (Barker, 2014, Mowat and Agace, 2014). Similar to both the small intestine and colon linings, the caecal epithelium is generated by the division of long-lived intestinal stem cells (ISC) that reside near the bottom of the crypts and produce proliferating transit-amplifying (TA) progenitor cells that later differentiate, giving rise to absorptive enterocytes and secretory cells (Paneth, goblet, enteroendocrine and tuft cells) (Barker, 2014). However, the cellular composition of the caecal epithelium is different from that of the small intestine because in the caecum, goblet cells are numerous and found throughout the crypts while Paneth cells are rare (Mowat and Agace, 2014). The colon epithelium presents even larger numbers of goblet cells compared with the caecum but Paneth cells are absent (Mowat and Agace, 2014, Nguyen et al., 2015). This differential cellular composition contributes to variations in the thickness of the mucus layers overlaying the epithelium and in the microbiota structure (McGuckin et al., 2011, Mowat and Agace, 2014, James et al., 2020). These differences result in distinct niches that are colonised by enteric pathogens, which have successfully evolved to invade and persist in particular intestinal segments.

Understanding the embryonic development of the intestine and the signalling pathways that govern ISC proliferation and differentiation has enabled three-dimensional (3D) organoid cultures to be developed from small intestine and colon adult ISC (Sato et al., 2009, Sato et al., 2011, Sato and Clevers, 2013, Date and Sato, 2015). Organoids are capable of self-renewal and spatial organisation, and exhibit similar cellular composition, tissue architecture and organ functionality to their tissue of origin (Date and Sato, 2015, Fatehullah et al., 2016, Li and Izpisua Belmonte, 2019). Culture conditions for enteroids recreate the stem cell niche (SCN), including an extracellular matrix support that mimics the basal membrane component, and a combination of growth factors and morphogens (R-spondin 1, epidermal growth factor (EGF) and Noggin) that stimulate or inhibit the signalling pathways regulating ISC proliferation and differentiation (Sato et al., 2009, Sato and Clevers, 2013, Date and Sato, 2015). A gradient of Wingless-related integration site (Wnt) signalling, from Paneth cells, is required for the budding of crypt-like structures. The bottom of crypts contains stem and Paneth cells that push proliferating TA cells towards the lumen, where decreasing Wnt levels trigger terminal differentiation of the cells (Sato and Clevers, 2013). Wnt-producing Paneth cells are absent in the colon, so exogenous addition of Wnt ligand (Wnt3A) is required to maintain ISC division in colonoid cultures (Sato et al., 2011, Sato and Clevers, 2013, Date and Sato, 2015). However, the addition of Wnt3A to the medium causes the Wnt gradient to be lost and the organoids to become symmetric round cysts, consisting of a homogeneous population of stem and TA progenitor cells (Sato et al., 2011, Sato and Clevers, 2013). Thus, differentiation of colon organoids into crypt-like structures containing the different epithelial cell lineages requires the withdrawal of Wnt3A (Sato et al., 2011, Sato and Clevers, 2013).

**Fig. 1:**
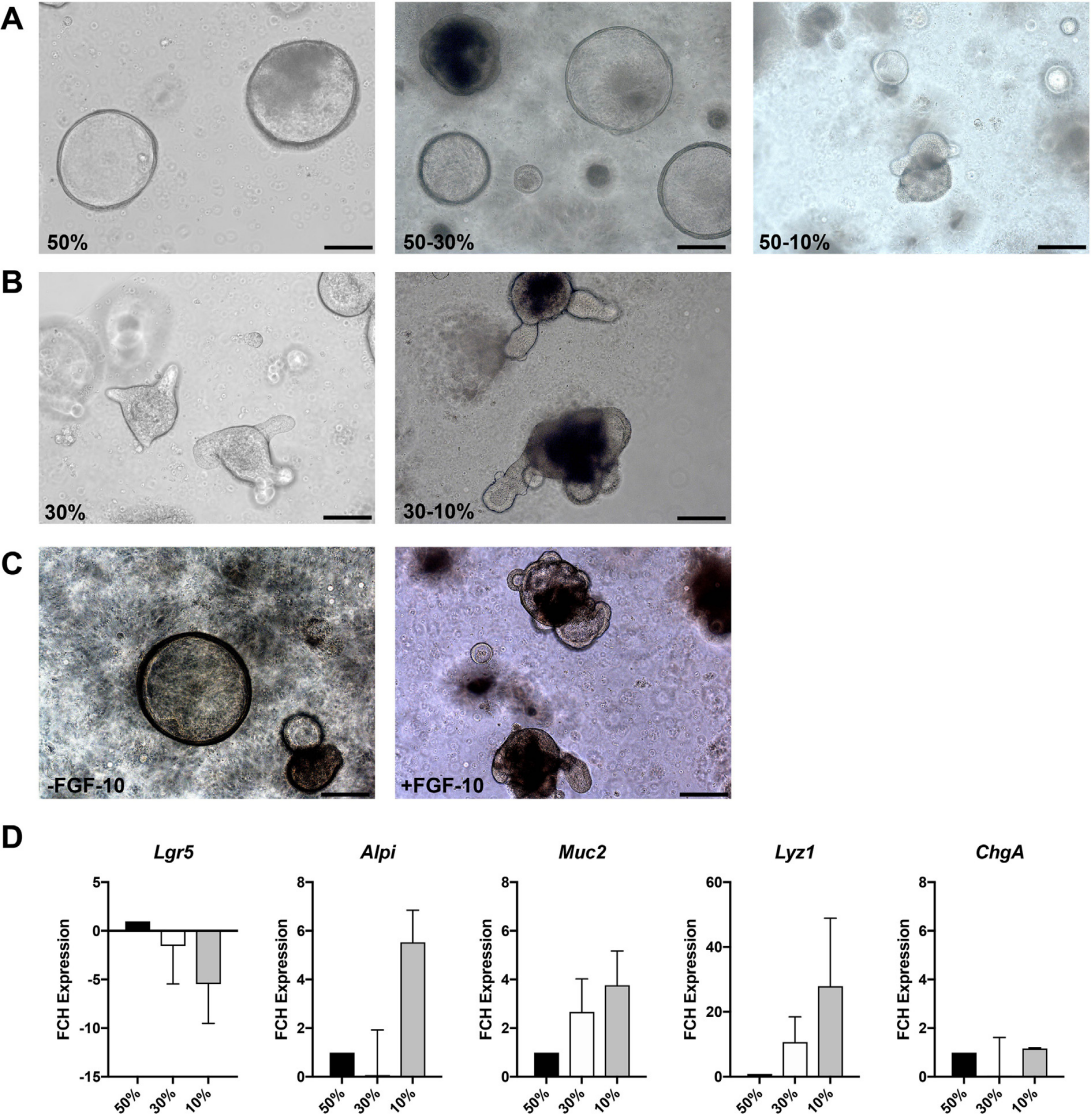
Murine caecaloid culture and differentiation. Representative bright field microscopy images of caecaloids grown in the presence of 50% (A, cystic-undifferentiated morphology) or 30% Wnt3A-conditioned medium (B, budding-differentiated morphology) and differentiated by reduction of concentration to 10%. (C) Images of caecaloids grown in 30% Wnt3A-conditioned medium in the absence (cystic-undifferentiated) or presence (budding-differentiated) of fibroblast growth factor-10. Scale bars = 200 μm. (D) Expression of marker genes measured by quantitative real-time-PCR for stem cells (*Lgr5*), enterocytes (*Alpi*), goblet cells (*Muc2*), Paneth cells (*Lyz1*) and enteroendocrine cells (*ChgrA*) in caecaloids grown in 50% and 30% Wnt3A-conditioned medium, and further differentiated by reduction of Wnt3A-conditioned medium from 30% to 10%. Results show the mean with S.D. of results from two different caecaloid lines. FCH, fold change.

Caecal organoid cultures, hereafter named caecaloids, have been generated before using similar culture conditions to those used for colonoids and similarly grow as symmetric round cysts (Miyoshi and Stappenbeck, 2013, Zaborin et al., 2017). However, upon withdrawal of Wnt3A, caecaloids do not recreate the differentiated budding crypt-like structures (Fig. 1A). Therefore, an alternative cocktail of growth factors/morphogens is needed to produce caecaloids that showcase the differentiated cell types and 3D spatial organisation present in the caecum.

The caecal epithelium is the primary colonisation site and port of entry for many clinically important pathogens for which mouse models exist, including *Trichuris trichiura* (model organism *T. muris), Salmonella enterica,* serovar Typhymurium, *Campylobacter jejuni, Shigella sonnei, Escherichia coli* (ETEC, model organism *Citrobacter rodentium*), *Yersinia pseudotuberculosis* and *Entamoeba histolytica,* among others (Lee et al., 1986, Pongpech et al., 1989, Houpt et al., 2002, Barthel et al., 2003, Klementowicz et al., 2012, Collins et al., 2014, Fahlgren et al., 2014). Developing mouse caecaloid cultures will enable host interactions of these important pathogens to be studied in an in vitro model. Ensuring that these organoids recapitulate the tissue architecture and contain the different IEC types present in the caecum is pivotal to the success of this model system.

Here, we established culture conditions for the long-term expansion, differentiation and characterisation of caecaloid cultures from adult mouse caecal ISC. Caecaloids closely recapitulated the full complement of stem and differentiated cell types present in the caecum, reproducing cellular composition differences between the caecal and small intestinal epithelium. To exemplify the use of caecaloids in the study of host–pathogen interactions in the caecum, we investigated the responses of caecaloids to extracellular vesicles (EVs) present in the excretory/secretory (ES) products of the mouse whipworm *T. muris*. EVs are lipid-enclosed structures that can deliver pathogen proteins and nucleic acids into host cells once internalised (Kuipers et al., 2018). *Trichuris muris* EVs can confer protection to whipworm infection in mice (Shears et al., 2018a) and one study has shown *T. muris* EVs are internalised by cells within colonoids (Eichenberger et al., 2018b). Here we examined the functional effects of *T. muris* EVs in caecaloids, which most closely match the in vivo context in which the parasites naturally reside. Using RNA sequencing (RNA-seq) of caecaloids microinjected with *T. muris* EVs we discovered a novel immune regulatory function of whipworm EVs on the caecal epithelium, namely the downregulation of responses to nucleic acid recognition and type-I IFN signalling. Our work provides a key tool for future analyses of host interactions with caecal pathogens and their products, and identifies new modulatory activities of helminth EVs on IECs.

## 2 Materials and methods

### 2.1 Enteroid and caecaloid culture

Enteroid and caecaloid lines from adult C57BL/6N mice (6–8 weeks old) were derived from small intestinal and caecal epithelial crypts. Briefly, tissues were cut open longitudinally and luminal contents removed. Tissues were then minced, segments were washed with ice cold Dulbecco’s PBS1X without calcium and magnesium (PBS) (Gibco Thermo Fisher Scientific, USA) and vigorous shaking to remove mucus, and treated with Gentle Cell Dissociation Reagent (STEMCELL Tech, UK) for 15 min at room temperature with continuous rocking. Released crypts were collected by centrifugation, washed with ice cold PBS, resuspended in 200 μl of cold Matrigel™ (Corning, USA), plated in 6-well tissue culture plates (Corning) and overlaid with a Wnt rich medium containing base growth medium (Advanced DMEM/F12 with 2 mM Glutamine, 10 mM HEPES, 1× penicillin/streptomycin (pen/strep), 1× B27 supplement, 1× N2 supplement (all from Gibco Thermo Fisher Scientific)), 50% Wnt3A conditioned medium (Wnt3A cell line, kindly provided by the Clevers laboratory, Utrecht University, Netherlands), 10% R-spondin1 conditioned medium (293 T-HA-Rspo1-Fc cell line, Trevigen, USA), 1 mM N-acetylcysteine (Sigma-Aldrich, UK), 50 ng/ml of rmEGF (Gibco Thermo Fisher Scientific), 100 ng/ml of rmNoggin (Peprotech, UK), 10 μM Rho kinase (ROCK) inhibitor (Y-27632) dihydrochloride monohydrate (Sigma-Aldrich), and exclusively for caecaloids, 100 ng/ml of rh fibroblast growth factor (FGF)-10 (Peprotech). Organoids were cultured at 37 °C, 5% CO_2_. The medium was changed every 2 days and after 1 week, pen/strep was removed from the medium and Wnt3A conditioned medium was completely removed for enteroids or reduced to 30% for caecaloids (expansion medium). Expanding enteroids and caecaloids were passaged, after recovering from Matrigel using ice-cold PBS or Cell Recovery Solution (Corning), by physical dissociation through vigorous pipetting with a p200 pipette every 5–7 days.

For differentiation of caecaloids, after passaging, organoids were grown in expansion medium for at least 2 days to allow reformation and growth in size. Then, medium was changed to differentiation medium containing 10% Wnt3A conditioned medium which was replaced every 2 days for up to 4 days.

### 2.2 Whole mount immunofluorescence staining of organoids

For whole mount staining, differentiated organoids were recovered from Matrigel using ice-cold PBS, re-plated onto chamber slides (Millicell EZ SLIDE 8-well glass, Millipore, USA) and cultured for an additional 2 days with differentiation medium. On the day of staining, organoids were fixed with 4% Formaldehyde, Methanol-free (Thermo Fisher, USA) in PBS for 1 h at room temperature, washed three times with PBS and permeabilized with 2% Triton X-100 (Sigma-Aldrich) 5% Foetal Bovine Serum (FBS) (Gibco Thermo Fisher Scientific) in PBS for 2 h at room temperature. Organoids were then incubated with primary antibodies α-villin (1:100, Abcam, UK, ab130751), α-Lysosyme (1:40, Dako, Agilent, USA A0099), α-Ki-67 (1:250, Abcam, ab16667), α-Chromogranin A (1:50, Abcam, ab15160), α-Dcamkl-1 (1:200, Abcam, ab31704) and the lectins *Ulex europaeus* agglutinin (UEA, 1:100, Sigma-Aldrich, 19337) and *Sambucus nigra* (SNA, 1:50, Vector Laboratories, UK, FL-1301) diluted in 0.25% Triton X-100 5% FBS in PBS overnight at 15 °C. After three washes with PBS, organoids were incubated with secondary antibody (Donkey anti-rabbit 555, 1:400, Molecular Probes, USA, A31572) for 6 h at room temperature or overnight at 15 °C. Organoids were washed three times with PBS and stained with DiIC18(5); 1,1′-dioctadecyl-3,3,3′,3′-tetramethylindodicarbocyanine, 4-chlorobenzenesulfonate salt (DiD, 2 µg/ml, Biotium, USA, 60014) overnight at 15 °C. After three washes with PBS, organoids were counterstained with DAPI (1:1,000, AppliChem, Germany, A1001.0010) at room temperature for 1 h. Organoids were washed six times with PBS and incubated with FocusClear™ (CelExplorer Labs Co, Taiwan) at room temperature for 1–2 h. Chamber slides were disassembled and mounted using ProLong Gold anti-fade reagent (Life Technologies Thermo Fisher Scientific, USA) and coverslips. Confocal microscopy images were taken with Leica SP8 (Germany) and LSM 510 Meta Zeiss (Germany) confocal microscopes, and processed using the Leica Application Suite X (LAS X) software.

### 2.3 Transmission electron microscopy (TEM)

Caecaloids were fixed in 2.5% glutaraldehyde/2% paraformaldehyde in 0.1 M sodium cacodylate buffer, post-fixed with 1% osmium tetroxide and mordanted with 1% tannic acid, followed by dehydration through an ethanol series (contrasting with uranyl acetate at the 30% stage) and embedding with an Epoxy Resin Kit (all from Sigma-Aldrich). Ultrathin sections cut on a Leica UC6 ultramicrotome were contrasted with uranyl acetate and lead nitrate, and images recorded on a FEI 120 kV Spirit Biotwin microscope (USA) on an F416 Tietz CCD camera.

### 2.4 Cell composition analysis of tissues and organoids using ImageStream

**Supplementary Fig. S1:**
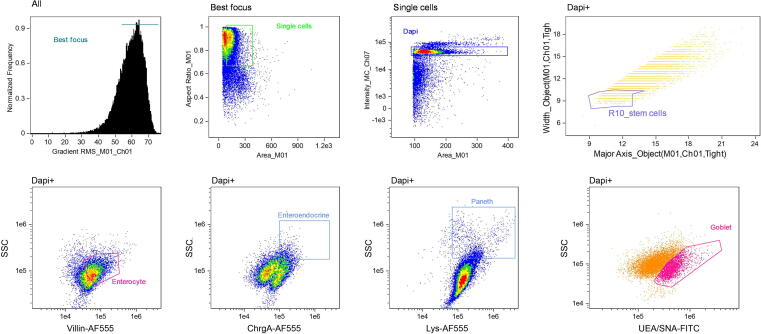
Gating strategy for ImageStream analysis. Gates were set up to select for single, in-focus cells using the guided analysis tools for focused and single cell analysis from Image Data Exploration and Analysis Software (IDEAS). Specifically, normalised frequency versus Gradient root mean square (RMS) on bright field) (BF) for all events was used to reject out-of-focus events and draw the Best focus gate. On the Best focus population, Aspect ratio versus Area for BF was used to exclude beads and select for single cell events (Single cells gate). “Single cells” were further gated based on nuclear stain to select for optimum nuclear staining by looking at Intensity for emission channel (Ch) 7 versus Area for BF (Dapi gate). All further analysis to quantify epithelial cell populations was performed using “Dapi gate” as the initial population. Intensity Ch6 (SSC) versus Ch3 (AF555) or Intensity Ch2 (FITC) was used for determining enterocyte, enteroendocrine, Paneth and goblet cell populations. To measure the stem cell population, the Features Finder tool from IDEAS was used, with Width Object versus Major Axis Object on BF used for gating.

Small intestines and caeca of mice were processed individually in parallel. Tissues were open longitudinally, washed with ice cold Hank’s Balanced Salt solution (HBSS) 1× (Gibco Thermo Fisher Scientific) containing 1× pen/strep to remove the luminal contents and cut into small fragments. These fragments were incubated at 37 °C in DMEM High Glucose (Gibco Thermo Fisher Scientific), 20% FBS, 2% Luria Broth, 1× pen/strep, 100 µg/ml of Gentamicin, 10 µM ROCK inhibitor and 0.5 mg/ml Dispase II (Sigma) with horizontal shaking for 90 min to detach epithelial crypts. The crypts containing supernatant were filtered through a 300 µm cell strainer (PluriSelect, Germany) and pelleted by centrifugation at 150*g* for 5 min at room temperature. Crypts and enteroids/caecaloids were dissociated into single cells by TrypLE Express (Gibco Thermo Fisher Scientific) digestion 10–20 min at 37 °C. The epithelial single-cell suspension was filtered through a 30 µm cell strainer (Sysmex, UK), washed and counted. Cells were fixed with 4% Formaldehyde, Methanol-free in PBS for 20 min at 4 °C, washed three times with PBS 1% FBS and permeabilized with 1× Perm/Wash solution (diluted from BD Perm/Wash Buffer 5× (USA) in PBS) at room temperature for 15 min. Cells were then incubated with the primary antibodies used for immunofluorescence staining diluted in 1× Perm/Wash solution for 30 min at 4 °C. Cells were washed three times with 1× Perm/Wash solution and stained with secondary antibody/lectins and DAPI diluted in 1× Perm/Wash solution for 30 min at 4 °C. After washes with 1× Perm/Wash solution and PBS, cells were resuspended in PBS. Samples were acquired on an Amnis ImageStream MkII Imaging Flow Cytometer (Luminex) at a low speed/high sensitivity flow rate and object magnification at 60× using the INSPIRE software. Data were analysed using the Image Data Exploration and Analysis Software (IDEAS) software. The gating strategy is shown in Supplementary Fig. S1.

### 2.5 RNA extraction and quantitative Real-Time PCR (qRT-PCR)

Caecaloids were recovered from Matrigel using Cell Recovery Solution and washed with ice-cold PBS. Caecaloids were then lysed with RTL buffer (RNeasy Mini-kit, QUIAGEN, Germany) plus beta-mercaptoethanol (Sigma-Aldrich) and RNA was extracted following the manufacturer’s instructions. Gene expression was quantified by quantitative real-time (qRT)-PCR using an ABsolute QPCR Mix, ROX and TaqMan primers (all from Thermo Fisher Scientific) for *Lgr5* (Mm00438890_m1), *Alpi* (Mm01285814_g1), *Muc2* (Mm01276696_m1), *Lyz1* (Mm00657323_m1), *Chga (*Mm00514341_m1) and *Gapdh* (Mm99999915_g1) in a StepOne Real Time PCR system (Applied Biosystems, USA).

### 2.6 Trichuris muris EV purification and quality control

*Trichuris muris* EVs were purified from the ES products of *T. muris* as previously described (Shears et al., 2018a). Briefly, approx. ~6000 adult parasites were cultured in RPMI medium supplemented with 500 U/ml of penicillin and 500 μg/ml of streptomycin (Sigma Aldrich) for 18 h (after removing the first 4 h of ES products). The ES products were spun at 720 *g* for 15 min to remove eggs and the supernatant was then filtered using a 0.22 μm filter (Millipore) to further remove debris. Supernatants were then ultra-centrifuged at 100,000 *g* for 2 h in polyallomer tubes (Beckman Coulter, USA) using an SV323 rotor. The ultracentrifuge pellet was washed with PBS and re-pelleted by a subsequent spin at 100,000 *g* for 2 h. The EV pellet was resuspended in 6 ml of PBS and stored at −80 °C prior to further concentration using a 5 kDa MW cut-off vivaspin (Sartorius, Germany). The protein content of the concentrated EVs was quantified using Qubit (Invitrogen, USA) as 0.18 μg/μl (a total yield of 34 μg of EVs for this batch) and the EV count was determined by Nanosight (Malvern Panalytical, UK) to be 2.1 × 10^7^/μl of EVs. EVs were diluted 2:3 with phenol red (Sigma Aldrich) (final concentration of 0.12 μg/μl) prior to microinjection into the organoids.

### 2.7 Microinjection of caecaloids

For microinjection, differentiated caecaloids were recovered from Matrigel using ice-cold PBS, re-plated onto microinjection plates (MatTek Corporation, USA) and cultured for an additional 2 days with differentiation medium. Microinjections were performed using the Eppendorf TransferMan NK2-FemtoJet express system (Germany), in an environmental chamber integrated to a Zeiss Axiovert 200 M bright field microscope, to allow all injections to be carried out at 37 °C and 5% CO_2_. The injector settings were a pressure of 400 kPa and an injection time of 0.5 s. For microinjections, Piezo Drill Tip Mouse ICSI (6 μm) (Eppendorf) microcapillaries were used. For RNA sequencing (RNA-seq), 50 caecaloids per microinjection plate were injected with either PBS (as control) or EVs diluted with phenol red so that injected caecaloids could be easily identified. After injection, caecaloids were incubated for 24 h at 37 °C, 5% CO_2_, recovered using Cell Recovery Solution and total RNA was extracted as described above.

### 2.8 RNA-seq and analysis

RNA-seq was performed in organoids microinjected with PBS or *T. muris* EVs (*n* = 3) in technical triplicates. Multiplexed cDNA libraries were generated from high quality RNA samples (RNA integrity number ≥7.0) according to the Illumina TruSeq RNA Preparation protocol, and sequenced on an Illumina HiSeq platform. We obtained 3.9–4.5 million paired end reads per sample; raw data have been submitted to ENA (European Nucleotide Archive, https://www.ebi.ac.uk/ena) under the following accessions: ERS2914946, ERS2914953, ERS2914962, ERS2914970, ERS2914978, ERS2914986. Kallisto (v0.43.1) (Bray et al., 2016) was used to pseudoalign reads to the mouse GRCm38 transcriptome (downloaded from Ensembl release 97(Yates et al., 2020), https://www.ensembl.org), with over 92% of reads per sample pseudoaligning. For differential expression analysis, the DESeq function from the DESeq2 package (v1.24.0) (Love et al., 2014) was used to fit a negative binomial generalised lineal model for each gene and estimate log2 fold changes, and *P* values were calculated with a Wald test. Genes with an adjusted *P* value <0.05 are reported as being differentially expressed. Innate DB v5.4 (Breuer et al., 2013) (https://www.innatedb.com) was used to identify enriched Gene Ontology (GO) terms. The variance stabilising transformation function was used to transform counts for Principal Component Analysis (PCA) and heat map plotting. For inclusion in the heatmap, genes were selected based on an association with viral response related GO terms (GO:0051607, GO:0009615, GO:0098586, GO:0039536) by Innate DB and/or Ensembl and an absolute log2fold change >1.

## 3 Results

### 3.1 Establishment of 3D caecaloid culture conditions

The caecum, similar to other parts of the intestine, is composed of two layers: (i) an internal endoderm-derived columnar epithelium with absorptive and secretory functions and; (ii) an external surrounding mesoderm-derived mesenchyme (Burns et al., 2004, Al Alam et al., 2012). The mouse caecum develops as a bud propagating off the main gut tube early in the differentiation of the gastrointestinal tract (from day 10.5 of embryonic development) (Burns et al., 2004). Epithelial-mesenchymal interactions are critical for the formation of gastrointestinal buds such as the caecum and the stomach. In particular, FGF-10, expressed specifically in the mesenchyme of the caecal bud, signals via the FGF receptor 2b of the epithelium to promote epithelial proliferation at the caecal bud during days 10.5–14.5 of embryonic development (Burns et al., 2004, Zhang et al., 2006, Al Alam et al., 2012)*.* Stomach organoid cultures require FGF-10, in addition to the mouse colonoid culture conditions, to drive budding events and expansion of the cultures (Barker et al., 2010). With this in mind, and considering the presence of small numbers of Paneth cells in the caecum (Mowat and Agace, 2014, Nguyen et al., 2015) that contribute Wnt to the culture, we modified existing protocols for colonoid generation (Sato et al., 2011), by adjusting the concentration of Wnt3A in culture medium and including FGF-10.

Upon isolation, crypts were cultured in 50% Wnt3A-conditioned medium until organoids were formed, presenting a cystic morphology (Fig. 1A). After the first passage, the Wnt3A-conditioned medium concentration was reduced to 30% for long-term maintenance culture. At this concentration and in the presence of FGF-10, caecaloids grew as a mixture of cystic and budding organoids, and were easily committed to full differentiation by a reduction of Wnt3A-conditioned medium concentration to 10% (Fig. 1B and C). FGF-10 was critical to drive budding events on caecaloids (Fig. 1C), just as in stomach organoids (Barker et al., 2010). In the absence of Wnt3A (enteroid culture condition) caecaloids did not survive, indicating the requirement of Wnt3A addition to the medium for their expansion (data not shown). However, when long-term culturing caecaloids with 50% Wnt3A-conditioned medium, as required for the culture of colonoids, it was not possible to induce their differentiation by withdrawal of Wnt3A (Fig. 1A). These results indicate caecal ISC have specific growth factor requirements for division and differentiation, which are modelled in vitro by fine-tuning the addition of exogenous Wnt ligands and mimicking epithelial-mesenchymal interactions by addition of FGF-10.

### 3.2 Differentiated caecaloids closely recapitulate the caecal epithelium

While budding morphology is a sign of differentiation of organoids, we next sought to evaluate if differentiation culture conditions (initial culture after passaging in expansion medium with 30% Wnt3A-conditioned medium for 2 days, followed by 4 days culture in differentiation medium with 10% Wnt3A-conditioned medium) resulted in full epithelial maturation of caecaloids, recreating the caecal epithelium. Therefore, to characterise the caecaloid cellular composition, we first used qRT-PCR to evaluate the expression of known IEC populations markers including Leucine-rich repeat-containing G-protein coupled receptor 5 (*Lgr5)* for stem cells, alkaline phosphatase (*Alpi*) for absorptive enterocytes, mucin 2 (*Muc2*) for goblet cells, lysozyme 1 (*Lyz1*) for Paneth cells and chromogranin A (*ChgA*) for enteroendocrine cells (Fig. 1D). Gene expression was measured in caecaloids maintained with 50% or 30% Wnt3A-conditioned medium and in differentiated caecaloids cultured as described above. All markers were detected, confirming the presence of all cellular populations. When comparing caecaloids grown in the presence of 50% and 30% Wnt3A-conditioned medium, we observed a downregulation of *Lgr5* in the latter group, indicating a reduction in the number of stem cells (Fig. 1D). Conversely, growing organoids under expansion conditions (30% Wnt3A) induced the expression of *Muc2* and *Lyz1*, suggesting an increase in goblet and Paneth cells, respectively (Fig. 1D). Upon differentiation of caecaloids by decreasing the Wnt3A-conditioned medium concentration to 10%, we observed further downregulation of *Lgr5* and detected upregulation of *Alpi,* indicating an increase in absorptive enterocytes (Fig. 1D). These results support our morphological observations (Fig. 1B) of further differentiation of organoids upon reduction of the concentration of Wnt3A in the culture medium (Fig. 1B).

**Fig. 2:**
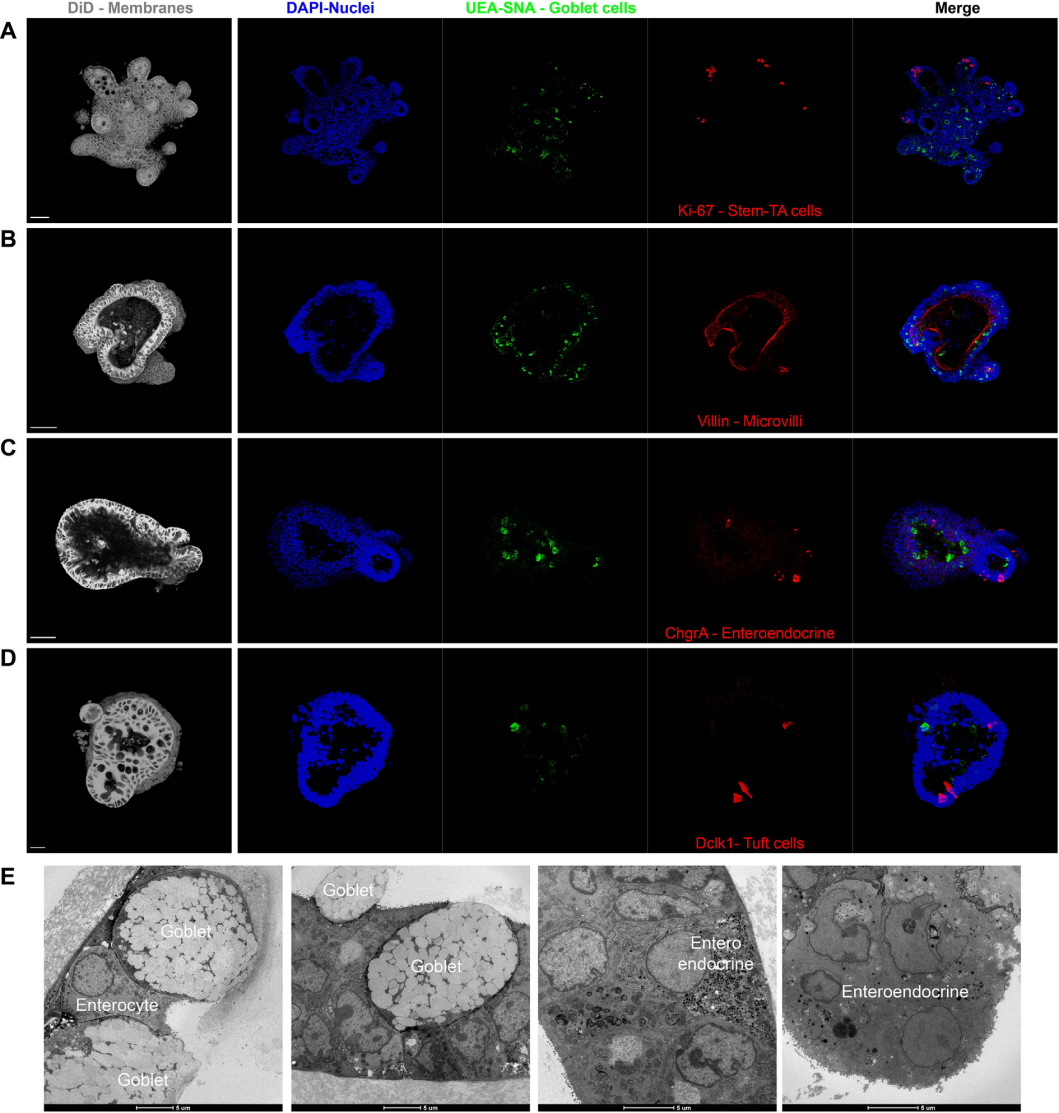
Confocal microscopy characterisation of differentiated murine caecaloids. Images of caecaloids expanded and further differentiated by reduction of Wnt3A-conditioned medium from 30% to 10%, showing the presence of all Intestinal Epithelial Cell populations. (A–D) Confocal immunofluorescence microscopy with antibody staining (A) Ki-67, marker of proliferating cells, stem and transit-amplifying cells; (B) villin, identifying microvilli of enterocytes; (C) chromogranin A expressing enteroendocrine cells; (D) Dclk-1, marker of tuft cells; and with the lectins *Ulex europaeus agglutinin* and *Sambucus nigra* that bind mucus in goblet cells. DAPI stains nuclei and DiIC18(5); 1,1′-dioctadecyl-3,3,3′,3′-tetramethylindodicarbocyanine, 4-chlorobenzenesulfonate salt the cell membranes. Scale bar = 50 μm for (A, B, C and E), 20 μm for (D). (E) Transmission Electron Microscopy images showing enterocytes, goblet and enteroendocrine cells present in caecaloids.

**Fig. 3:**
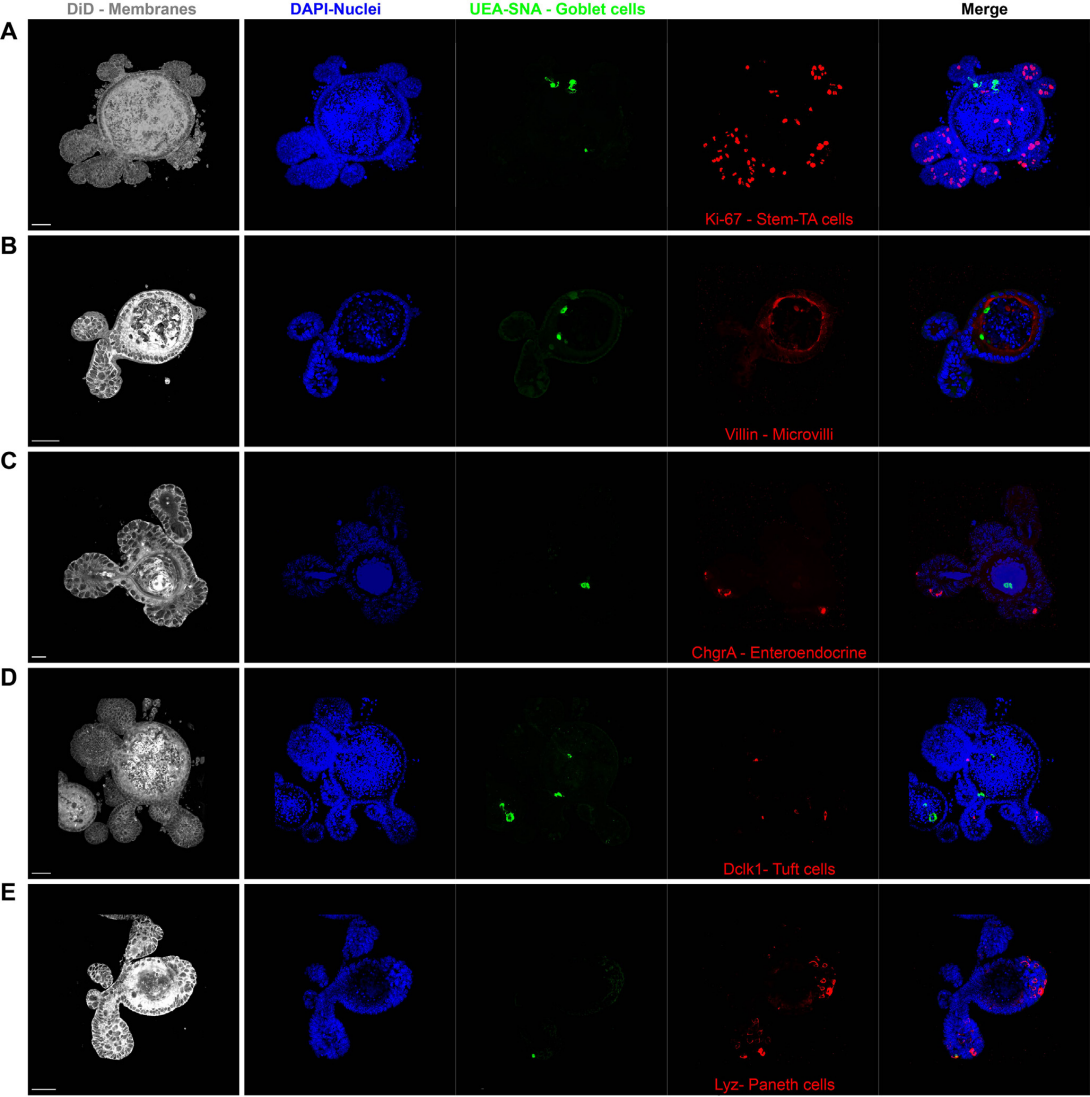
Confocal microscopy characterisation murine enteroids. Confocal immunofluorescence microscopy images of enteroids, showing the presence of all Intestinal Epithelial Cell populations stained with antibodies for (A) Ki-67, marker of proliferating cells, stem and transit-amplifying cells; (B) villin, identifying microvilli of enterocytes; (C) chromogranin A expressing enteroendocrine cells; (D) Dclk-1, marker of tuft cells; (E) lysozyme expressing Paneth cells; and with the lectins *Ulex europaeus agglutinin and Sambucus nigra* that bind mucus in goblet cells. DAPI stains nuclei and DiIC18(5); 1,1′-dioctadecyl-3,3,3′,3′-tetramethylindodicarbocyanine, 4-chlorobenzenesulfonate salt the cell membranes. Scale bar = 50 μm for (A, B, D and E), 20 μm for (C).

**Supplementary Fig. S2:**
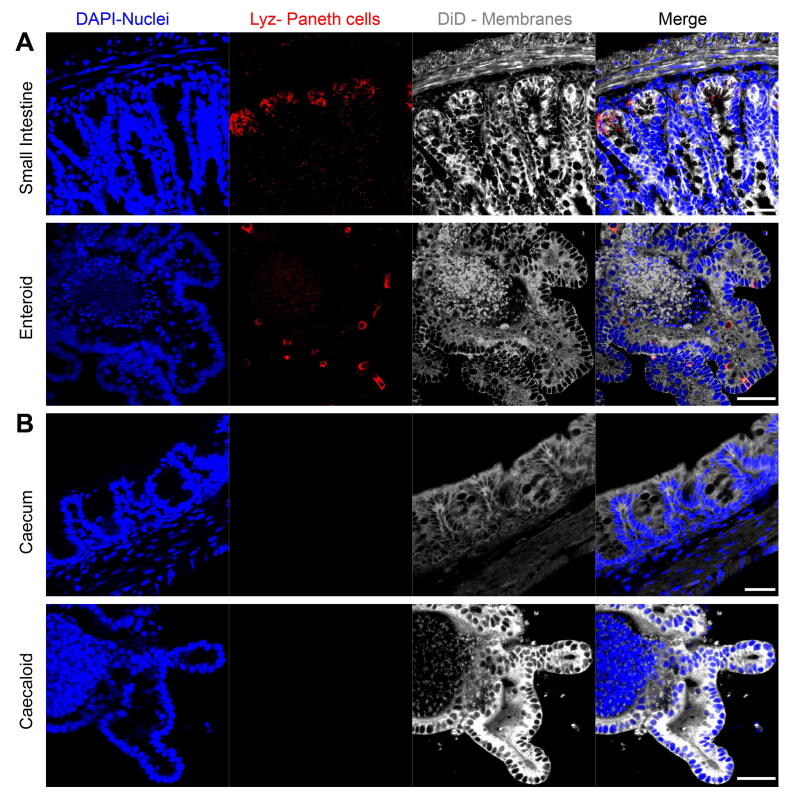
Lysozyme staining as a marker of Paneth cells in murine small intestine and caecum tissue and organoids. Images of confocal IF microscopy with an antibody staining Lysozyme expressing Paneth cells in small intestine tissue and enteroids (A), which are not frequently found in caecal tissue and caecaloids (B). DAPI stains nuclei and DiIC18(5); 1,1′-dioctadecyl-3,3,3′,3′-tetramethylindodicarbocyanine, 4-chlorobenzenesulfonate salt (DiD) the cell membranes. Scale bar = 100 μm.

To further study the differentiation status of the caecaloids, we performed confocal immunofluorescence microscopy of caecaloids and enteroids (Fig. 2, Fig. 3). The cellular markers analysed were Ki-67 (present in proliferating stem and TA cells), villin (staining microvilli on absorptive enterocytes), chromogranin A (marker of enteroendocrine cells), lysozyme (produced by Paneth cells), Dclk-1 (identifying tuft cells), and a combination of the lectins UEA and SNA that bind mucus on goblet cells. We observed that differentiated caecaloids contained the following: proliferating cells (Ki-67^+^) at the bottom of budding regions (Fig. 2A), microvilli of enterocytes bordering the lumen (Fig. 2B), a few enteroendocrine (Fig. 2C) and tuft cells (Fig. 2D) but numerous goblet cells (Fig. 2A–D). The presence of enterocytes, goblet and enteroendocrine cells in caecaloids was confirmed using TEM (Fig. 2E). We did not detect Paneth cells in caecaloids using this methodology (Supplementary Fig. S2). In contrast, enteroids have numerous Paneth cells (Fig. 3E) present at the bottom of budding regions where Ki-67^+^ cells are also located. Enteroids have fewer goblets cells (Fig. 3A–E), and show similar levels of enteroendocrine (Fig. 3C) and tuft cells (Fig. 3D) compared with caecaloids (Fig. 2).

**Fig. 4:**
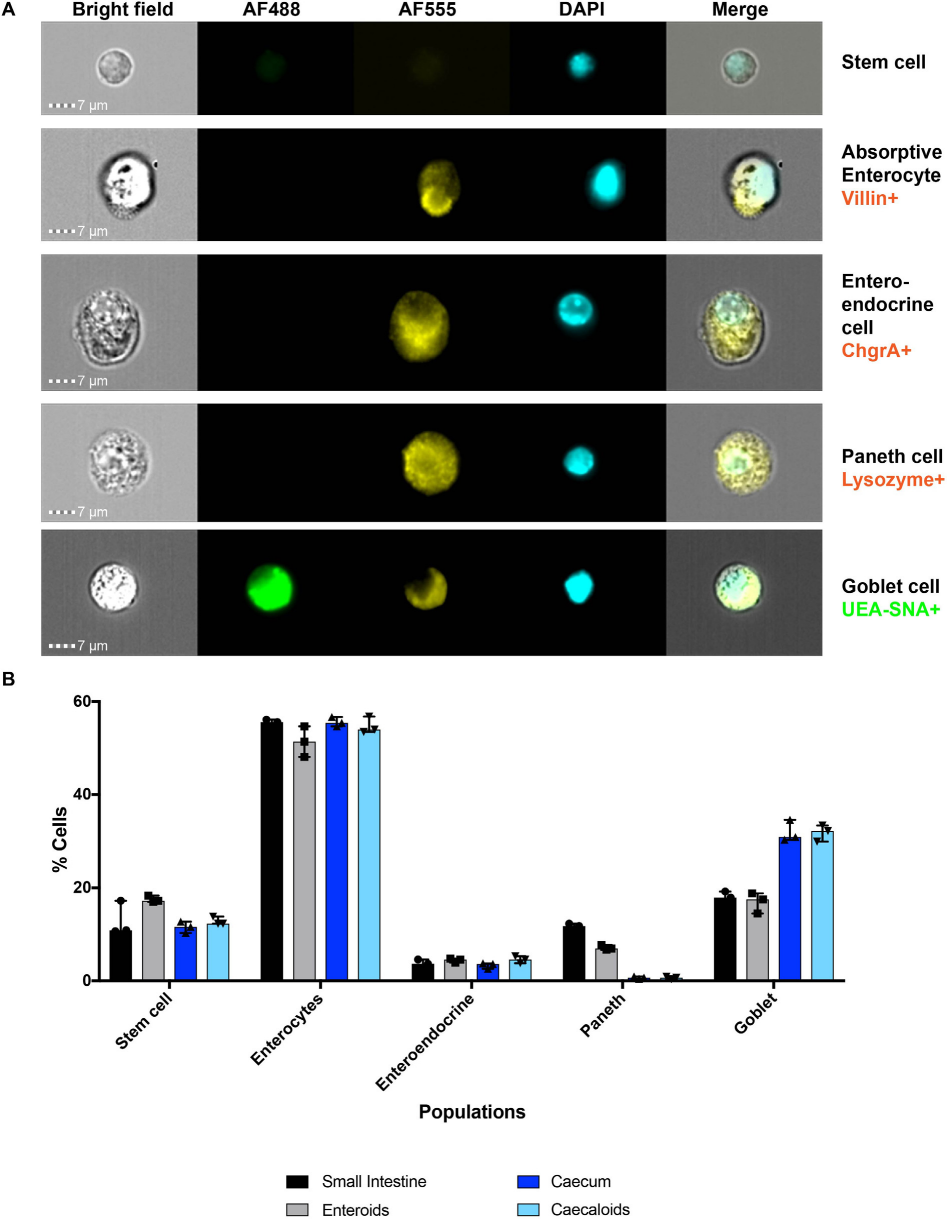
Comparison of cellular composition of murine enteroids, caecaloids and small intestine and caecum tissues by ImageStream. Enteroids, caecaloids and Intestinal Epithelial Cells from small intestine and caecum were dissociated into single cells, stained with antibodies and lectins targeting enterocytes, enteroendocrine, Paneth and goblet cells, and visualised by ImageStream. (A) Bright field and fluorescence representative images of cellular populations. Scale bar = 7 μm. (B) Percentages (median with interquartile range) of cellular populations identified by ImageStream. *n* = 3. Note the consistency on the composition of organoids and the tissue of origin.

Next, we aimed to determine how caecaloids and enteroids reflect the cellular composition of the tissue of origin. Thus, to quantitatively characterise the different cellular populations in tissue and organoids, we performed ImageStream analysis on single cell preparations stained with antibodies and lectins for markers of the major IEC populations. ImageStream combines both bright field and fluorescence microscopy, coupled with flow cytometry capabilities, allowing enterocytes as well as stem, enteroendocrine, Paneth and goblet cells to be clearly identified and quantified (Fig. 4A and Supplementary Fig. S1). We found a remarkable similarity in the percentages of the different cell types in the organoids and the tissue from which they were derived (Fig. 4B). Moreover, these results confirmed our observations using confocal immunofluorescence staining that showed proportionally more goblet cells in the caecal tissue and caecaloids than in the small intestine and in enteroids (Fig. 4B). Conversely, the proportions of Paneth cells are lower in the caecum and caecaloids compared with the small intestine and enteroids (Fig. 4B). The proportions of enterocytes, enteroendrocrine and stem cells are similar among both tissues and organoids (Fig. 4B). Together these data demonstrate that our methods allow the generation of caecaloids, closely recapitulating the cellular composition and architecture of the caecal epithelium.

### 3.3 Caecaloids as a model to study host–pathogen interactions: understanding the effects of *T. muris* EVs on caecal IECs

**Fig. 5:**
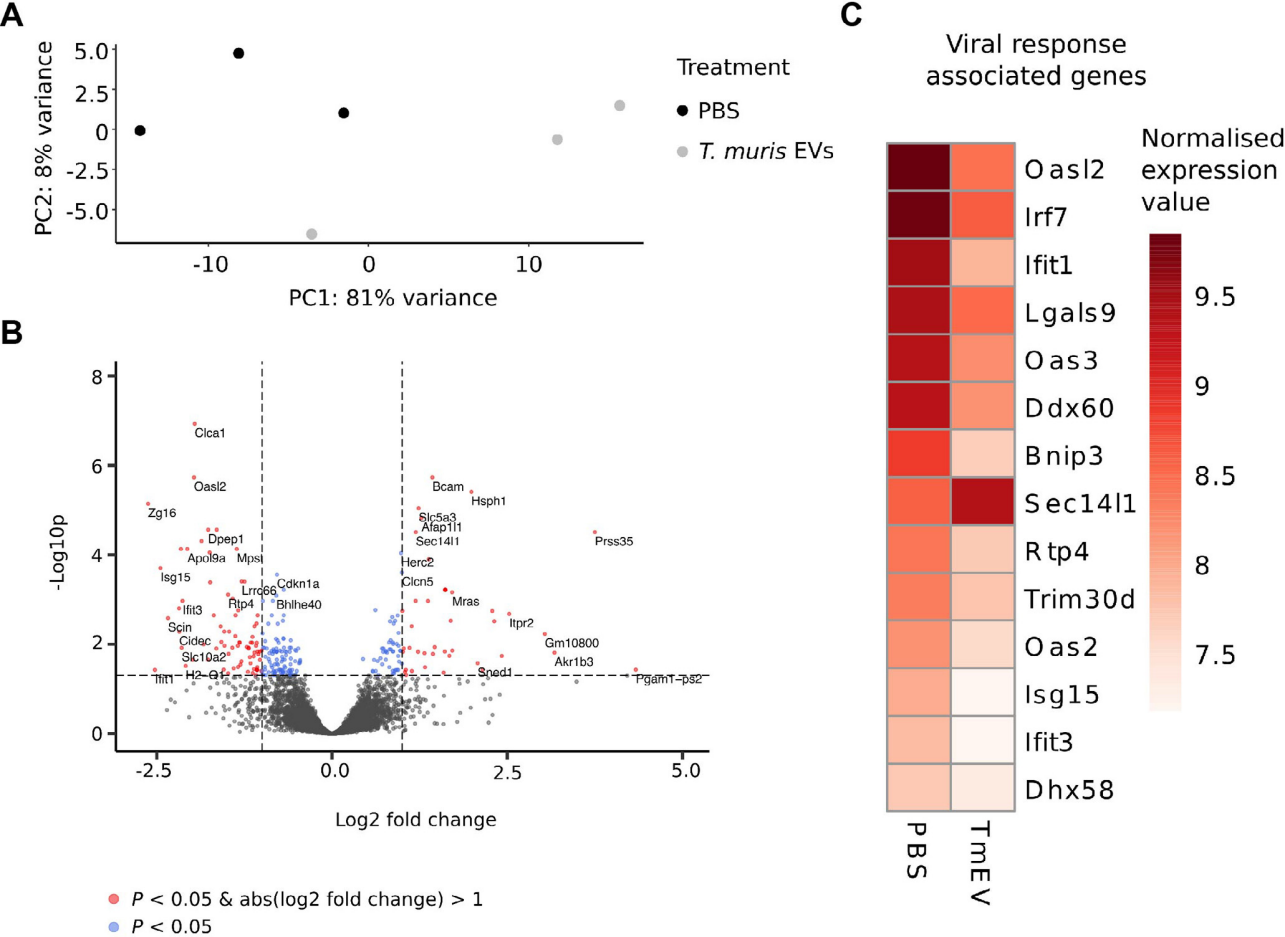
Transcriptional response of murine caecaloids to microinjection of *Trichuris muris* extracellular vesicles. (A) Principal component analysis showing sample clustering across PC1 and PC2. (B) Volcano plot showing transcriptional response to microinjection of *T. muris* EVs. Genes significantly differentially expressed (adjusted *P* value <0.05) are indicated in red (absolute log2 fold change >1) or blue (absolute log2 fold change <1). (C) Heat map of transformed and normalised expression counts for selected genes (mean of three replicates is represented). Viral response-associated genes are associated with the Gene Ontology terms GO:0051607 and/or GO:0009615 by Innate DB (https://www.innatedb.com/) and/or Ensembl (https://www.ensembl.org).

**Supplementary Fig. S3:**
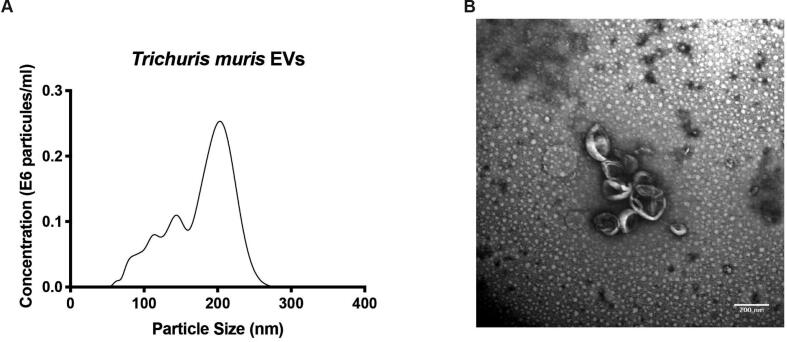
Qualitative analysis of *Trichuris muris* extracellular vesicles (EVs) by (A) Transmission Electron Microscopy and (B) profile of EVs by Nanosight, at 1:1,000 dilution.

Next, we aimed to use caecaloids as an in vitro model to study interactions between pathogens invading the caecum and the epithelium of this organ. Whipworms are intracellular helminths that inhabit the caecal epithelium. In order to persist in their host, whipworms modulate intestinal inflammation, ensuring their host’s and their own survival (Klementowicz et al., 2012, Grencis, 2015). One such mechanism of immunomodulation is the release of ES products that can interact with immune cells, the regulatory impact of which has been described (Kuijk et al., 2012, Klaver et al., 2013, Laan et al., 2017, Leroux et al., 2018, Shears et al., 2018b, Bancroft et al., 2019). ES products likely act also on caecal IECs, given that whipworms live inside the epithelium; however, little is known about these interactions (Hiemstra et al., 2014). One component of whipworm ES products is EVs, lipid membrane enclosed structures with the capacity to transfer a multitude of nucleic acids and proteins to a single cell at once, which have been shown to be potent host modulators (Buck et al., 2014, Hansen et al., 2015, Coakley et al., 2017, Tritten et al., 2017, Eichenberger et al., 2018a, Eichenberger et al., 2018b, Shears et al., 2018a). To date, the responses of caecal IECs to whipworm EVs have not been studied. To investigate these interactions, we purified EVs from the ES products of *T. muris* adult worms (Supplementary Fig. S3) and microinjected the EVs into caecaloids. As the apical surface of the IECs in 3D caecaloids is facing the lumen (see microvilli (Villin) staining Fig. 2B), microinjection is therefore required to mimic the interactions that naturally take place in the caecum (Duque-Correa et al., 2020). PBS was microinjected in 3D caecaloids as a control. After 24 h of culture, total RNA was extracted and gene expression changes in response to EV administration were evaluated by RNA-seq. We observed a clear response of the caecaloids to the EVs (Fig. 5A and B) with a total of 88 genes upregulated and 173 genes downregulated. Interestingly, stimulation with EVs secreted by adult *T. muris* parasites resulted in significantly reduced expression of viral response-associated genes by caecal IECs (Fig. 5C). Specifically, we detected decreased expression of genes involved in the cytosolic sensing of nucleic acids including *Dhx58, Ddx60* and *Irf7*, which are part of the signalling cascade that results upon engagement of retinoic-acid inducible gene I (RIG-I)-like receptors by double-stranded RNA (dsRNA) (Liu et al., 2016). Consequently, EV treatment of caecaloids resulted in downregulation of interferon stimulated genes (ISGs) comprising *Oasl2*, *Oas2* and *3, Ifit1* and *3*, *and Isg15,* which are transcribed in response to nucleic acid recognition and type-I IFN signalling (Perng and Lenschow, 2018). Although this is just one example of the downstream use of caecaloids, our results suggest the anti-inflammatory effects of whipworm infections and their ES products can be, at least in part, mediated by a direct effect on the caecal epithelium.

## 4 Discussion

Here we developed culture conditions for the long-term maintenance and differentiation of caecaloids closely resembling the composition and spatial conformation of the caecal epithelium. Our methods fill a critical gap in the protocols to generate organoids, recreating the differences on the epithelium that distinguish all intestinal segments and that are crucial in the use of these in vitro systems to model host–enteric pathogen interactions.

Several pathogens have a tropism for the caecum and the particularities of its mucosa provide a defined niche for which these bacteria and parasites have evolved mechanisms to invade and colonise. In particular, whipworms are large metazoan parasites that live in the caecum of their host, where they tunnel inside IECs, creating a multi-intracellular niche (Tilney et al., 2005, Klementowicz et al., 2012). Whipworms can remain in their host for years, causing chronic infections. To optimise their residence in their hosts, whipworms manipulate host inflammation, partly through the immunoregulatory effects of ES products released by the parasites (Kuijk et al., 2012, Klaver et al., 2013, Hansen et al., 2015, Laan et al., 2017, Tritten et al., 2017, Eichenberger et al., 2018b, Leroux et al., 2018, Shears et al., 2018a, Shears et al., 2018b, Bancroft et al., 2019). Progress has been made in understanding the composition and anti-inflammatory actions of whipworm ES products. This has largely involved proteomic analyses of the ES products and, more recently, characterisation of the protein and nucleic acid cargo of EVs (Hansen et al., 2015, Tritten et al., 2017, Leroux et al., 2018, Shears et al., 2018a, Shears et al., 2018b, Eichenberger et al., 2018b, White et al., 2020). Moreover, immunomodulatory effects of adult *T. suis* (natural pig whipworm) and *T. muris* ES products on different immune cells have been described (Kuijk et al., 2012, Klaver et al., 2013, Laan et al., 2017, Leroux et al., 2018, Bancroft et al., 2019). In contrast, very little is understood regarding the modulatory functions of ES products, or EVs in particular, on IECs, with only one report showing that *T. suis* ES stimulation of an epithelial cell line results in reduced barrier function and decreased lipopolysaccharide-induced TNF-α and CXCL1 production (Hiemstra et al., 2014). IECs are important sensors of intestinal helminth infections, initiating innate immune responses and with specialised effector functions that contribute to parasite expulsion (Artis and Grencis, 2008, Grencis, 2015). Compared with cell lines, organoids more accurately reproduce the composition and architecture of the intestinal epithelium and recently have been used to characterise the interactions and IEC responses to ES products of various helminths (Duque-Correa et al., 2019). Specifically, stimulation of murine enteroids with *Trichinella spiralis* ES products and extracts indicated that sensing of parasitic products by tuft-cell receptors results in Ca^2+^ responses (Luo et al., 2019). Moreover, imaging experiments of murine enteroids and colonoids microinjected with EVs present in the ES products of *Nippostrongylus brasiliensis* and *T. muris,* respectively*,* showed their uptake by host IECs (Eichenberger et al., 2018a, Eichenberger et al., 2018b). A similar approach has been used to visualise the internalisation of *Ascaris suum* EVs co-cultured with canine enteroids (Chandra et al., 2019). However, to date organoids have not been exploited to study host IEC responses to helminth EVs.

Here, for the first known time, we evaluated the functional effects of helminth EVs on IECs using organoids. Microinjection of adult *T. muris* EVs in fully differentiated caecaloids resulted in downregulation of expression of viral response-associated genes by caecal IECs, including those involved in cytosolic sensing of nucleic acids via RIG-I-like receptors, and ISGs produced in response to nucleic acid recognition and type-I IFN signalling (Liu et al., 2016, Perng and Lenschow, 2018). Intriguingly, *T. muris* EVs contain parasite small RNAs (sRNAs) (Tritten et al., 2017, Eichenberger et al., 2018b, White et al., 2020) which, instead of triggering host responses to foreign RNA, appear to supress such detection mechanisms. This suppression may enable EV regulatory functions as it allows foreign RNA cargo to operate without being sensed by the host cell. Recent publications have shown type-I IFN responses are induced in response to helminth infections. In particular, stimulation with *Schistosoma mansoni* antigens (Webb et al., 2017) and infection with *N. brasiliensis* (Connor et al., 2017) results in type-I IFN signalling in dendritic cells, which is required for initiation of Th2 responses. In the setting of *Heligmosomoides polygyrus* infection, type-I IFN responses are reported to be upregulated in the duodenum (McFarlane et al., 2017) and inhibit granuloma formation around larval parasites (Reynolds et al., 2014). Interestingly, type-I IFN responses have not been previously associated with whipworm infections, but their relevance to the development of type 2 immunity in other helminths suggests that adult whipworms may suppress type-I IFN responses via EVs to counteract host immune responses that result in their expulsion. Our findings open, therefore, a new avenue of investigation on the interactions of the worm with its host cells and the role of IECs as sensors and orchestrators of the immune responses against whipworms. In the near future, we aim to understand the mechanisms by which the nucleic acids and protein cargo of the EVs exert such functions in caecaloids. In this regard, our studies on the sRNA composition of *T. muris* EVs presented in this special issue (White et al., 2020) will help enable identification of targets in the host IECs. At this stage we do not know whether there are multiple *T. muris* EV subsets present in our purification and future work could focus on further purifying the subsets that are specifically responsible for suppressing the type-I IFN response. Moreover, our methods for microscopy characterisation of IEC populations in caecaloids will be pivotal to pinpoint IEC types that preferentially internalise EVs and their intracellular interactions. These future experiments will also shed light on the immunoregulatory effects of therapies using live parasitic worms including whipworms *(T. trichiura* and *T. suis)*, worm secretions and worm-derived synthetic molecules that are being trialled to treat inflammatory gastrointestinal diseases (Smallwood et al., 2017, Varyani et al., 2017). On a broader note, future comparative studies analysing the uptake and functional responses of enteroids, caecaloids and colonoids to EVs from different intestinal helminths will ascertain if EVs are specialised to specific niches and could also illuminate differences in epithelial cell responses from the different intestinal segments.

The remarkable recapitulation of the caecal epithelium achieved by caecaloids will allow the interactions of other caecal pathogens and commensals with the IECs of this organ to be studied with precision. In particular, the multicellularity of this in vitro system can be exploited to investigate the role of different IEC populations in pathogen invasion and colonisation, host damage and responses (Duque-Correa et al., 2019). The up- and down-regulation of cell populations and factors in a caecal-specific context can also be evaluated after exposure to pathogens and their products (Duque-Correa et al., 2019). In addition, caecaloids can be used in studies investigating how caecal microbiota impact the caecal epithelium composition and metabolism. Moreover, caecaloids could be used to model inflammatory pathologies of the caecum, including cancer and Inflammatory Bowel Disease, and better understand their aetiology and compare it with inflammation present in other intestinal segments.

In the future, complementation of caecaloid cultures with other tissue components including cellular populations of the SCN (stromal and immune cells), commensal microbiota, chemical gradients and physical/mechanical forces (Fatehullah et al., 2016, Barrila et al., 2018; Duque-Correa et al., 2019; Takebe and Wells, 2019) will more closely recreate the caecal native microenvironment and provide a more complex model to investigate caecal pathologies and the intricacies of pathogen mechanisms to colonise and modulate these niches.

## References

[b0005] Al Alam D., Sala F.G., Baptista S., Galzote R., Danopoulos S., Tiozzo C., Gage P., Grikscheit T., Warburton D., Frey M.R., Bellusci S. (2012). FGF9-Pitx2-FGF10 signaling controls cecal formation in mice. Dev. Biol..

[b0010] Artis D., Grencis R.K. (2008). The intestinal epithelium: sensors to effectors in nematode infection. Mucosal Immunol..

[b0015] Backhed F., Ley R.E., Sonnenburg J.L., Peterson D.A., Gordon J.I. (2005). Host-bacterial mutualism in the human intestine. Science.

[b0020] Bancroft A.J., Levy C.W., Jowitt T.A., Hayes K.S., Thompson S., McKenzie E.A., Ball M.D., Dubaissi E., France A.P., Bellina B., Sharpe C., Mironov A., Brown S.L., Cook P.C., A, S.M., Thornton, D.J., Grencis, R.K (2019). The major secreted protein of the whipworm parasite tethers to matrix and inhibits interleukin-13 function. Nat. Commun..

[b0025] Barker N. (2014). Adult intestinal stem cells: critical drivers of epithelial homeostasis and regeneration. Nat. Rev. Mol. Cell Biol..

[b0030] Barker N., Huch M., Kujala P., van de Wetering M., Snippert H.J., van Es J.H., Sato T., Stange D.E., Begthel H., van den Born M., Danenberg E., van den Brink S., Korving J., Abo A., Peters P.J., Wright N., Poulsom R., Clevers H. (2010). Lgr5(+ve) stem cells drive self-renewal in the stomach and build long-lived gastric units in vitro. Cell Stem Cell.

[b0035] Barrila J., Crabbe A., Yang J., Franco K., Nydam S.D., Forsyth R.J., Davis R.R., Gangaraju S., Ott C.M., Coyne C.B., Bissell M.J., Nickerson C.A. (2018). Modeling host-pathogen interactions in the context of the microenvironment: three-dimensional cell culture comes of age. Infect. Immun..

[b0040] Barthel M., Hapfelmeier S., Quintanilla-Martinez L., Kremer M., Rohde M., Hogardt M., Pfeffer K., Russmann H., Hardt W.D. (2003). Pretreatment of mice with streptomycin provides a Salmonella enterica serovar Typhimurium colitis model that allows analysis of both pathogen and host. Infect. Immun..

[b0045] Bray N.L., Pimentel H., Melsted P., Pachter L. (2016). Near-optimal probabilistic RNA-seq quantification. Nat. Biotechnol..

[b0050] Breuer K., Foroushani A.K., Laird M.R., Chen C., Sribnaia A., Lo R., Winsor G.L., Hancock R.E., Brinkman F.S., Lynn D.J. (2013). InnateDB: systems biology of innate immunity and beyond–recent updates and continuing curation. Nucleic Acids Res..

[b0055] Buck A.H., Coakley G., Simbari F., McSorley H.J., Quintana J.F., Le Bihan T., Kumar S., Abreu-Goodger C., Lear M., Harcus Y., Ceroni A., Babayan S.A., Blaxter M., Ivens A., Maizels R.M. (2014). Exosomes secreted by nematode parasites transfer small RNAs to mammalian cells and modulate innate immunity. Nat. Commun..

[b0060] Burns R.C., Fairbanks T.J., Sala F., De Langhe S., Mailleux A., Thiery J.P., Dickson C., Itoh N., Warburton D., Anderson K.D., Bellusci S. (2004). Requirement for fibroblast growth factor 10 or fibroblast growth factor receptor 2-IIIb signaling for cecal development in mouse. Dev. Biol..

[b0065] Chandra L., Borcherding D.C., Kingsbury D., Atherly T., Ambrosini Y.M., Bourgois-Mochel A., Yuan W., Kimber M., Qi Y., Wang Q., Wannemuehler M., Ellinwood N.M., Snella E., Martin M., Skala M., Meyerholz D., Estes M., Fernandez-Zapico M.E., Jergens A.E., Mochel J.P., Allenspach K. (2019). Derivation of adult canine intestinal organoids for translational research in gastroenterology. BMC Biol..

[b0070] Coakley G., McCaskill J.L., Borger J.G., Simbari F., Robertson E., Millar M., Harcus Y., McSorley H.J., Maizels R.M., Buck A.H. (2017). Extracellular vesicles from a helminth parasite suppress macrophage activation and constitute an effective vaccine for protective immunity. Cell Rep.

[b0075] Collins J.W., Keeney K.M., Crepin V.F., Rathinam V.A., Fitzgerald K.A., Finlay B.B., Frankel G. (2014). Citrobacter rodentium: infection, inflammation and the microbiota. Nat. Rev. Microbiol..

[b0080] Connor L.M., Tang S.C., Cognard E., Ochiai S., Hilligan K.L., Old S.I., Pellefigues C., White R.F., Patel D., Smith A.A., Eccles D.A., Lamiable O., McConnell M.J., Ronchese F. (2017). Th2 responses are primed by skin dendritic cells with distinct transcriptional profiles. J. Exp. Med..

[b0085] Date S., Sato T. (2015). Mini-gut organoids: reconstitution of the stem cell niche. Annu. Rev. Cell Dev. Biol..

[b0090] Duque-Correa M.A., Maizels R.M., Grencis R.K., Berriman M. (2020). Organoids - new models for host-helminth interactions. Trends Parasitol..

[b0095] Eckburg P.B., Bik E.M., Bernstein C.N., Purdom E., Dethlefsen L., Sargent M., Gill S.R., Nelson K.E., Relman D.A. (2005). Diversity of the human intestinal microbial flora. Science.

[b0100] Eichenberger R.M., Ryan S., Jones L., Buitrago G., Polster R., Montes de Oca M., Zuvelek J., Giacomin P.R., Dent L.A., Engwerda C.R., Field M.A., Sotillo J., Loukas A. (2018). Hookworm secreted extracellular vesicles interact with host cells and prevent inducible colitis in mice. Front. Immunol..

[b0105] Eichenberger R.M., Talukder M.H., Field M.A., Wangchuk P., Giacomin P., Loukas A., Sotillo J. (2018). Characterization of *Trichuris muris* secreted proteins and extracellular vesicles provides new insights into host-parasite communication. J. Extracell. Vesic..

[b0110] Fahlgren A., Avican K., Westermark L., Nordfelth R., Fallman M. (2014). Colonization of cecum is important for development of persistent infection by Yersinia pseudotuberculosis. Infect. Immun..

[b0115] Fatehullah A., Tan S.H., Barker N. (2016). Organoids as an in vitro model of human development and disease. Nat. Cell Biol..

[b0120] Grencis R.K. (2015). Immunity to helminths: resistance, regulation, and susceptibility to gastrointestinal nematodes. Annu. Rev. Immunol..

[b0125] Hansen E.P., Kringel H., Williams A.R., Nejsum P. (2015). Secretion of RNA-Containing Extracellular Vesicles by the Porcine Whipworm, *Trichuris suis*. J. Parasitol..

[b0130] Hiemstra I.H., Klaver E.J., Vrijland K., Kringel H., Andreasen A., Bouma G., Kraal G., van Die I., den Haan J.M. (2014). Excreted/secreted *Trichuris suis* products reduce barrier function and suppress inflammatory cytokine production of intestinal epithelial cells. Mol. Immunol..

[b0135] Houpt E.R., Glembocki D.J., Obrig T.G., Moskaluk C.A., Lockhart L.A., Wright R.L., Seaner R.M., Keepers T.R., Wilkins T.D., Petri W.A. (2002). The mouse model of amebic colitis reveals mouse strain susceptibility to infection and exacerbation of disease by CD4+ T cells. J. Immunol..

[b0140] James K.R., Gomes T., Elmentaite R., Kumar N., Gulliver E.L., King H.W., Stares M.D., Bareham B.R., Ferdinand J.R., Petrova V.N., Polanski K., Forster S.C., Jarvis L.B., Suchanek O., Howlett S., James L.K., Jones J.L., Meyer K.B., Clatworthy M.R., Saeb-Parsy K., Lawley T.D., Teichmann S.A. (2020). Distinct microbial and immune niches of the human colon. Nat. Immunol..

[b0145] Klaver E.J., Kuijk L.M., Laan L.C., Kringel H., van Vliet S.J., Bouma G., Cummings R.D., Kraal G., van Die I. (2013). *Trichuris suis*-induced modulation of human dendritic cell function is glycan-mediated. Int. J. Parasitol..

[b0150] Klementowicz J.E., Travis M.A., Grencis R.K. (2012). *Trichuris muris*: a model of gastrointestinal parasite infection. Sem. Immunopathol..

[b0155] Kuijk L.M., Klaver E.J., Kooij G., van der Pol S.M., Heijnen P., Bruijns S.C., Kringel H., Pinelli E., Kraal G., de Vries H.E., Dijkstra C.D., Bouma G., van Die I. (2012). Soluble helminth products suppress clinical signs in murine experimental autoimmune encephalomyelitis and differentially modulate human dendritic cell activation. Mol. Immunol..

[b0160] Kuipers M.E., Hokke C.H., Smits H.H., Nolte-'t Hoen E.N.M. (2018). Pathogen-derived extracellular vesicle-associated molecules that affect the host immune system: an overview. Front. Microbiol..

[b0165] Laan L.C., Williams A.R., Stavenhagen K., Giera M., Kooij G., Vlasakov I., Kalay H., Kringel H., Nejsum P., Thamsborg S.M., Wuhrer M., Dijkstra C.D., Cummings R.D., van Die I. (2017). The whipworm (*Trichuris suis*) secretes prostaglandin E2 to suppress proinflammatory properties in human dendritic cells. FASEB J..

[b0170] Lee A., O'Rourke J.L., Barrington P.J., Trust T.J. (1986). Mucus colonization as a determinant of pathogenicity in intestinal infection by *Campylobacter jejuni*: a mouse cecal model. Infect. Immun..

[b0175] Leroux L.P., Nasr M., Valanparambil R., Tam M., Rosa B.A., Siciliani E., Hill D.E., Zarlenga D.S., Jaramillo M., Weinstock J.V., Geary T.G., Stevenson M.M., Urban J.F., Mitreva M., Jardim A. (2018). Analysis of the *Trichuris suis* excretory/secretory proteins as a function of life cycle stage and their immunomodulatory properties. Sci. Rep..

[b0180] Li M., Izpisua Belmonte J.C. (2019). Organoids - preclinical models of human disease. N. Engl. J. Med..

[b0185] Liu Y., Olagnier D., Lin R. (2016). Host and viral modulation of RIG-I-mediated antiviral immunity. Front. Immunol..

[b0190] Love M.I., Huber W., Anders S. (2014). Moderated estimation of fold change and dispersion for RNA-seq data with DESeq2. Genome Biol..

[b0195] Luo X.C., Chen Z.H., Xue J.B., Zhao D.X., Lu C., Li Y.H., Li S.M., Du Y.W., Liu Q., Wang P., Liu M., Huang L. (2019). Infection by the parasitic helminth *Trichinella spiralis* activates a Tas2r-mediated signaling pathway in intestinal tuft cells. Proc. Natl. Acad. Sci. U. S. A..

[b0200] McFarlane A.J., McSorley H.J., Davidson D.J., Fitch P.M., Errington C., Mackenzie K.J., Gollwitzer E.S., Johnston C.J.C., MacDonald A.S., Edwards M.R., Harris N.L., Marsland B.J., Maizels R.M., Schwarze J. (2017). Enteric helminth-induced type I interferon signaling protects against pulmonary virus infection through interaction with the microbiota. J. Allergy Clin. Immunol..

[b0205] McGuckin M.A., Linden S.K., Sutton P., Florin T.H. (2011). Mucin dynamics and enteric pathogens. Nat. Rev. Microbiol..

[b0210] Miyoshi H., Stappenbeck T.S. (2013). In vitro expansion and genetic modification of gastrointestinal stem cells in spheroid culture. Nat. Protoc..

[b0215] Mowat A.M., Agace W.W. (2014). Regional specialization within the intestinal immune system. Nat. Rev. Immunol..

[b0220] Nguyen T.L., Vieira-Silva S., Liston A., Raes J. (2015). How informative is the mouse for human gut microbiota research?. Dis. Model. Mech..

[b0225] Perng Y.C., Lenschow D.J. (2018). ISG15 in antiviral immunity and beyond. Nat. Rev. Microbiol..

[b0230] Pongpech P., Hentges D.J., Marsh W.W., Eberle M.E. (1989). Effect of streptomycin administration on association of enteric pathogens with cecal tissue of mice. Infect. Immun..

[b0235] Reynolds L.A., Harcus Y., Smith K.A., Webb L.M., Hewitson J.P., Ross E.A., Brown S., Uematsu S., Akira S., Gray D., Gray M., MacDonald A.S., Cunningham A.F., Maizels R.M. (2014). MyD88 signaling inhibits protective immunity to the gastrointestinal helminth parasite *Heligmosomoides polygyrus*. J. Immunol..

[b0240] Sato T., Clevers H. (2013). Growing self-organizing mini-guts from a single intestinal stem cell: mechanism and applications. Science.

[b0245] Sato T., Stange D.E., Ferrante M., Vries R.G., Van Es J.H., Van den Brink S., Van Houdt W.J., Pronk A., Van Gorp J., Siersema P.D., Clevers H. (2011). Long-term expansion of epithelial organoids from human colon, adenoma, adenocarcinoma, and Barrett's epithelium. Gastroenterology.

[b0250] Sato T., Vries R.G., Snippert H.J., van de Wetering M., Barker N., Stange D.E., van Es J.H., Abo A., Kujala P., Peters P.J., Clevers H. (2009). Single Lgr5 stem cells build crypt-villus structures in vitro without a mesenchymal niche. Nature.

[b0255] Shears R.K., Bancroft A.J., Hughes G.W., Grencis R.K., Thornton D.J. (2018). Extracellular vesicles induce protective immunity against *Trichuris muris*. Parasite Immunol..

[b0260] Shears R.K., Bancroft A.J., Sharpe C., Grencis R.K., Thornton D.J. (2018). Vaccination against whipworm: identification of potential immunogenic proteins in *Trichuris muris* excretory/secretory material. Sci. Rep..

[b0265] Smallwood T.B., Giacomin P.R., Loukas A., Mulvenna J.P., Clark R.J., Miles J.J. (2017). Helminth immunomodulation in autoimmune disease. Front. Immunol..

[b0270] Takebe T., Wells J.M. (2019). Organoids by design. Science.

[b0275] Tilney L.G., Connelly P.S., Guild G.M., Vranich K.A., Artis D. (2005). Adaptation of a nematode parasite to living within the mammalian epithelium. J. Exp. Zool. A: Comp. Exp. Biol..

[b0280] Tritten L., Tam M., Vargas M., Jardim A., Stevenson M.M., Keiser J., Geary T.G. (2017). Excretory/secretory products from the gastrointestinal nematode *Trichuris muris*. Exp. Parasitol..

[b0285] Varyani F., Fleming J.O., Maizels R.M. (2017). Helminths in the gastrointestinal tract as modulators of immunity and pathology. Am. J. Physiol. Gastrointest. Liver Physiol..

[b0290] Webb L.M., Lundie R.J., Borger J.G., Brown S.L., Connor L.M., Cartwright A.N., Dougall A.M., Wilbers R.H., Cook P.C., Jackson-Jones L.H., Phythian-Adams A.T., Johansson C., Davis D.M., Dewals B.G., Ronchese F., MacDonald A.S. (2017). Type I interferon is required for T helper (Th) 2 induction by dendritic cells. EMBO J..

[b0295] White R., Kumar S., Chow F.W.N., Robertson E., Hayes K.S., Grencis R.K., Duque-Correa M.A., Buck A. (2020). Extracellular vesicles from *Heligmosomoides bakeri* and *Trichuris muris* contain distinct microRNA families and small RNAs that could underpin different functions in the host. Int. J. Parasitol..

[b0300] Yates A.D., Achuthan P., Akanni W., Allen J., Allen J., Alvarez-Jarreta J., Amode M.R., Armean I.M., Azov A.G., Bennett R., Bhai J., Billis K., Boddu S., Marugan J.C., Cummins C., Davidson C., Dodiya K., Fatima R., Gall A., Giron C.G., Gil L., Grego T., Haggerty L., Haskell E., Hourlier T., Izuogu O.G., Janacek S.H., Juettemann T., Kay M., Lavidas I., Le T., Lemos D., Martinez J.G., Maurel T., McDowall M., McMahon A., Mohanan S., Moore B., Nuhn M., Oheh D.N., Parker A., Parton A., Patricio M., Sakthivel M.P., Abdul Salam A.I., Schmitt B.M., Schuilenburg H., Sheppard D., Sycheva M., Szuba M., Taylor K., Thormann A., Threadgold G., Vullo A., Walts B., Winterbottom A., Zadissa A., Chakiachvili M., Flint B., Frankish A., Hunt S.E., Garth I.I., Kostadima M., Langridge N., Loveland J.E., Martin F.J., Morales J., Mudge J.M., Muffato M., Perry E., Ruffier M., Trevanion S.J., Cunningham F., Howe K.L., Zerbino D.R., Flicek P. (2020). Ensembl 2020. Nucleic Acids Res..

[b0305] Zaborin A., Krezalek M., Hyoju S., Defazio J.R., Setia N., Belogortseva N., Bindokas V.P., Guo Q., Zaborina O., Alverdy J.C. (2017). Critical role of microbiota within cecal crypts on the regenerative capacity of the intestinal epithelium following surgical stress. Am. J. Physiol. Gastrointest. Liver Physiol..

[b0310] Zhang X., Stappenbeck T.S., White A.C., Lavine K.J., Gordon J.I., Ornitz D.M. (2006). Reciprocal epithelial-mesenchymal FGF signaling is required for cecal development. Development.

